# Medical Wikis Dedicated to Clinical Practice: A Systematic Review

**DOI:** 10.2196/jmir.3574

**Published:** 2015-02-19

**Authors:** Alexandre Brulet, Guy Llorca, Laurent Letrilliart

**Affiliations:** ^1^Département de médecine généraleFaculté de Médecine Lyon EstUniversité Claude Bernard Lyon 1Lyon CEDEX 08France; ^2^Département de rhumatologieCentre Hospitalier Lyon SudPierre-BéniteFrance; ^3^Equipe d’Accueil 4129 « Santé Individu Société »Faculté de Médecine LaënnecUniversité de LyonLyonFrance

**Keywords:** wikis, clinical medicine, review

## Abstract

**Background:**

Wikis may give clinician communities the opportunity to build knowledge relevant to their practice. The only previous study reviewing a set of health-related wikis, without specification of purpose or audience, globally showed a poor reliability.

**Objective:**

Our aim was to review medical wiki websites dedicated to clinical practices.

**Methods:**

We used Google in ten languages, PubMed, Embase, Lilacs, and Web of Science to identify websites. The review included wiki sites, accessible and operating, having a topic relevant for clinical medicine, targeting physicians or medical students. Wikis were described according to their purposes, platform, management, information framework, contributions, content, and activity. Purposes were classified as “encyclopedic” or “non-encyclopedic”. The information framework quality was assessed based on the Health On the Net (HONcode) principles for collaborative websites, with additional criteria related to users’ transparency and editorial policy. From a sample of five articles per wikis, we assessed the readability using the Flesch test and compared articles according to the wikis’ main purpose. Annual editorial activities were estimated using the Google engine.

**Results:**

Among 25 wikis included, 11 aimed at building an encyclopedia, five a textbook, three lessons, two oncology protocols, one a single article, and three at reporting clinical cases. Sixteen wikis were specialized with specific themes or disciplines. Fifteen wikis were using MediaWiki software as-is, three were hosted by online wiki farms, and seven were purpose-built. Except for one MediaWiki-based site, only purpose-built platforms managed detailed user disclosures. The owners were ten organizations, six individuals, four private companies, two universities, two scientific societies, and one unknown. Among 21 open communities, 10 required users’ credentials to give editing rights. The median information framework quality score was 6 out of 16 (range 0-15). Beyond this score, only one wiki had standardized peer-reviews. Physicians contributed to 22 wikis, medical learners to nine, and lay persons to four. Among 116 sampled articles, those from encyclopedic wikis had more videos, pictures, and external resources, whereas others had more posology details and better readability. The median creation year was 2007 (1997-2011), the median number of content pages was 620.5 (3-98,039), the median of revisions per article was 17.7 (3.6-180.5) and 0.015 of talk pages per article (0-0.42). Five wikis were particularly active, whereas six were declining. Two wikis have been discontinued after the completion of the study.

**Conclusions:**

The 25 medical wikis we studied present various limitations in their format, management, and collaborative features. Professional medical wikis may be improved by using clinical cases, developing more detailed transparency and editorial policies, and involving postgraduate and continuing medical education learners.

## Introduction

 Access to information is a daily concern for clinicians, especially in general practice where the expertise field is particularly wide. Clinicians have to apply evidence-based knowledge as far as possible to manage varied and complex medical issues [1]. The medical information they use for practice must be accurate, readable, reliable, and up to date. As the use of primary sources requires documentary research methods and is time-consuming, clinicians usually refer to available syntheses such as practice guidelines, educational journals, or medical textbooks. However, these resources are often limited by language barriers [2], missing evidence [3], low acceptability [4], and conflicts of interest [5].

Wikis are websites characterized by a collaborative edition between users. A “wiki” is a type of content managing system differing from others in that the content is created without any defined owner [6]. Wikis belong to Web 2.0, which includes other interactive Web tools such as blogs (where users edit their own content), forums (where users discuss), and social networks (where users post comments) [7]. Since the wiki principle was initiated in 1995 on WikiWikiWeb, a site dedicated to programmers, hundreds of types of software have been developed to operate it [8]. Among them, MediaWiki is a worldwide reference that supports the 285 languages of the general encyclopedia Wikipedia. Subsequently, various medical wikis have emerged, including orphan diseases’ resources, terminology databases, care decision supports, and medical teaching resources [9-12]. Wikis may help to remediate other medical resources’ limitations by giving clinician communities the opportunity to build knowledge relevant to their practice [13].

The recent review of the literature about wikis and collaborative writing applications in health care by Archambault et al broadly explored use patterns, quality of information, and knowledge translation interests, and brought out a need for primary research on these applications [14]. Among the 25 articles in this review assessing the quality of the information, all but one targeted Wikipedia [15], whose medical content is controversial [16-18]. In the study published in 2009 by Dobrogowska-Schlebusch [15], 52 health-related wikis were included without specification of purpose or audience and assessed using the online Health Summit Working Group Information Quality tool (HSWG IQ tool) [19]. It globally showed poor quality scores, except for a few wikis having implemented expert moderation or peer reviews. The “quality of information” in a website actually refers either to its framework, including transparency and policy considerations such as in the HSWG IQ tool, or to its content, especially its scientific value. Assessing the content in wikis is problematic as it is only a snapshot of a long-lasting interaction [20-22].

Our study aimed at systematically reviewing medical wikis dedicated to clinical practices according to their purposes, platform, management, information framework, contributions, content, and activity.

## Methods

###  Screening Strategy

In October 2011, we performed Google queries searching for the phrase “list of medical wikis” translated in the 10 most spoken languages on the Internet (English, Chinese, Spanish, Japanese, French, Portuguese, German, Arabic, Russian, and Korean), using the Google translation tool when necessary [23]. The phrase was expanded as far as possible within the limit of 500 resulting pages. The English query was filtered in order to remove an extensively cited page, which has been kept once for data extraction [24]. Every resulting page was browsed in order to extract Internet addresses (uniform resource locators [URLs]) linking to potentially relevant sites (Multimedia Appendix 1).

Second, we searched PubMed and Web of Science (using “wiki” AND [“medic*” OR “clinic*”]) and Literatura Latino-Americana e do Caribe em Ciências da Saúde (LILACS) (using “wiki”) in full texts for articles published until September 2012. Every open-access abstract and open access article was read, coupled with Web searches when necessary, in order to identify any potentially relevant URL (Multimedia Appendix 2).

Finally, we included any other potentially relevant URL retrieved through Web extra-browsing or expert advice, until September 2012. One author (AB) made all data extractions of the screening.

###  Sites’ Inclusion and Exclusion

Websites were included if they were (1) accessible from a public Internet protocol address; (2) operating a wiki tool, defining a “wiki” as “a type of content managing system (CMS) used for collaborative edition, where the content is created without any defined owner” [6], excluding wiki-based platforms used as non-collaborative CMS, like Wikinu [25], and websites where a collaborative edition was allowed on owned contents, like Google Knols [26]; (3) aimed at building some knowledge relevant for a clinical practice, defining “clinical” as “of or relating to the bedside of a patient, the course of a disease, or the observation and treatment of patients directly” [27], excluding medical topics not directly linked to the care of patients (medical research, medical informatics, biomedical sciences, medical curriculums, pharmacology, public health), and topics not specifically interesting physicians (other health care disciplines, patient information, first aid); and (4) explicitly targeting physicians or medical students in audiences. Wikis orientated toward general public, like Wikipedia, were excluded [28]. In addition, websites were excluded if they were dysfunctional, explicitly interrupted, only aiming at displaying external resources. Some clinical-oriented wikis, like Medical Matters Wiki, were excluded as bibliographic resources [29].

The inclusion and exclusion was done by 2 authors (AB and LL), and disagreements were solved by discussion.

### Sites’ Description and Assessment

#### Overview

All data collections from the included sites were performed in October and November 2012. The main language interface of each wiki, that is, the one having the biggest amount of content, was used as a reference to collect data. No direct contact to sites’ administrators was undertaken. The data retrieval was done by 1 author (AB), and their assessments were performed by 2 authors (AB and LL). Disagreements were solved by discussion.

#### Purposes

Wikis’ main purposes were described on the basis of sites’ disclosures. Defining the term “encyclopedic” as a comprehensive reference work within a knowledge field [30], wikis were classified as “encyclopedic” or “non-encyclopedic” according to their statement of main purpose. Target audiences were described on the basis of sites’ disclosures, considering only physicians, medical students, and lay persons.

#### Platform

Platforms were described according to software, user data, ergonomics, and clinically relevant utilities, by systematically browsing sites and using their functionalities.

#### Management

Management was described on the basis of sites’ disclosures and technical characteristics. The access for editing was systematically tested anonymously and after login whenever registration was possible. A user community was defined as “closed” when the editing rights accreditation was not publicly opened. The registration process was defined as “automated” when filling out a form triggered the login access, and “on credentials” when some personal information had to be first checked. In case of hierarchy between registered users, those having special rights were consistently named “super-users”, and their nomination procedure and specific roles were described. We named “administrators” those super-users having enlarged rights such as deleting or massively editing content, assigning or removing rights to users, blocking pages, blocking users, etc.

#### Information Framework

The Health On the Net ethical code of conduct (HONcode), as adapted for collaborative websites, was used as a reference to perform the information framework quality assessment [31]. However, the adaptation of its principle about the authoritativeness of the information only makes mandatory the disclosure of the credentials of “moderators”. The wiki context makes every editing user responsible for edited content, and in a professional context, more author details than just credentials should be disclosed. We therefore built a set of 16 criteria for assessing the information framework quality, including 11 derived from the HONcode and 5 fitted to medical wikis. An operational definition was assigned to each of these criteria, including four definitions validated by Bernstam et al (Table 1) [32]. The assessment of these criteria was performed by 2 authors (AB and LL). Their agreement was measured by calculating an *r* correlation coefficient [33].

**Table 1:** The 16 information framework quality criteria.

Screened criteria^a^			Operational definition^b^
**Owner disclosures**			
	1	Identity (p2)	Indication of the entity that owns the information presented on the website (o1).
	2	Contact details (p6)	The webmaster or other official can be contacted. The presence of email address, telephone, fax, or online form (o2).
	3	Funding (p7)	The presence of a disclosure about owner’s funding.
	4	Conflicts of interest (p7)	The presence of a disclosure about owner’s conflicts of interest.
**Disclaimers**			
	5	Medical advisory statement (p2)	The presence of a statement about the value of the medical content displayed on the website.
	6	Users privacy policy (p3)	The presence of a disclosure about the management of the users’ personal information.
	7	Advertising policy (p8)	The presence of a disclosure about the advertising displayed (or not) in the website.
**Editorial policy**			
	8	Review policy (p1)	The presence of a claim of use of an editorial review process or the listing of an editorial review committee or medical advisory board (o3).
	9	Patients data protection rule (p3)	The presence of a rule for using patients’ data.
	10	Information referencing rule (p4)	The presence of a rule for referencing information.
	11	True statement rule (p5)	The presence of a rule for editing with honesty.
	12	Content organization rule	The presence of a rule for organizing the content.
**User disclosures**			
	13	Editing users’ identity	The presence of the disclosure of the identity, mandatory for every editing user.
	14	Editing users’ credentials	The presence of the disclosure of the authority and qualification (o4), mandatory for every editing user.
	15	Editing users’ conflicts of interest	The presence of the disclosure of eventual conflicts of interest, mandatory for every editing user.
	16	Administrators’ identity	The presence of the disclosure of the identity, mandatory for every administrator.

^a^Criteria referring to the HONcode principles [31]: p1=Information must be authoritative; p2=Purpose of the website; p3=Confidentiality; p4=Documented information; p5=Claims justification; p6=Website contact details; p7=Funding source disclosure; p8=Advertising policy.

^b^Operational definitions validated by Bernstam et al [32]: o1=Disclosure of ownership; o2=Feedback mechanism provided; o3=Editorial review process; o4=Author’s credentials disclosed.

#### Contributions

Physicians were considered as contributors by default, except when they were not targeted in the audience. The contributions of medical learners (students or physicians) were described based on educational objectives, or when mentioned in super-users’ credentials. Lay persons’ contributions were described according to the registration requirements. The presence of clinical case reports was systematically searched by querying sites with the key word “case”. Any content reporting some clinical materials issued from users’ practice was considered.

#### Content

This part of the study aimed at describing the characteristics of the contents and assessing their readability. However, the scientific value of contents in itself was not assessed. From each wiki, we selected a sample of the 5 most revised articles. Articles were included if they had a clinically relevant topic and were written in the main language of the wiki. In sites where the numbers of revisions were not available, we subjectively selected the most finalized articles. We described characteristics related to content (presence of pictures, videos, diagrams, posology details, evidence levels and external resources, and numbers of words and references per article) and data related to edition (numbers of revisions and authors per article, and related talks). The sampled articles were assessed with Flesch’s reading ease test adapted to each language and performed with automated hyphenation [34]. Characteristics of articles were compared between encyclopedic and non-encyclopedic groups by using Fisher’s exact test for qualitative data and the Wilcoxon rank test for quantitative data.

#### Activity

Wikis’ global activities were described on the basis of available data from sites (absolute numbers of content pages, revisions, and talk pages). Displayed numbers of users were considered globally inaccurate since we suspected tens of false user registrations across several sites, presumably due to vandalism attacks. In order to estimate annual activity, content pages were counted according to their last edition date by performing empty queries on Google, filtered on each URL, and for each year since the wiki’s creation. A recent editorial rate was estimated by reporting the number of pages last edited in the 365 previous days to that edited since creation. Rates higher than 50% were considered as “very high”, and rates lower than 10% were considered as “very low”. A recent editorial trend was estimated by reporting the number of pages last edited in the 365 previous days to that last edited in the 365 days before. Trends higher than 300% were considered as “sharply increasing” and trends lower than 33% as “sharply decreasing”.

## Results

### Sites’ Screening

**Figure 1:**
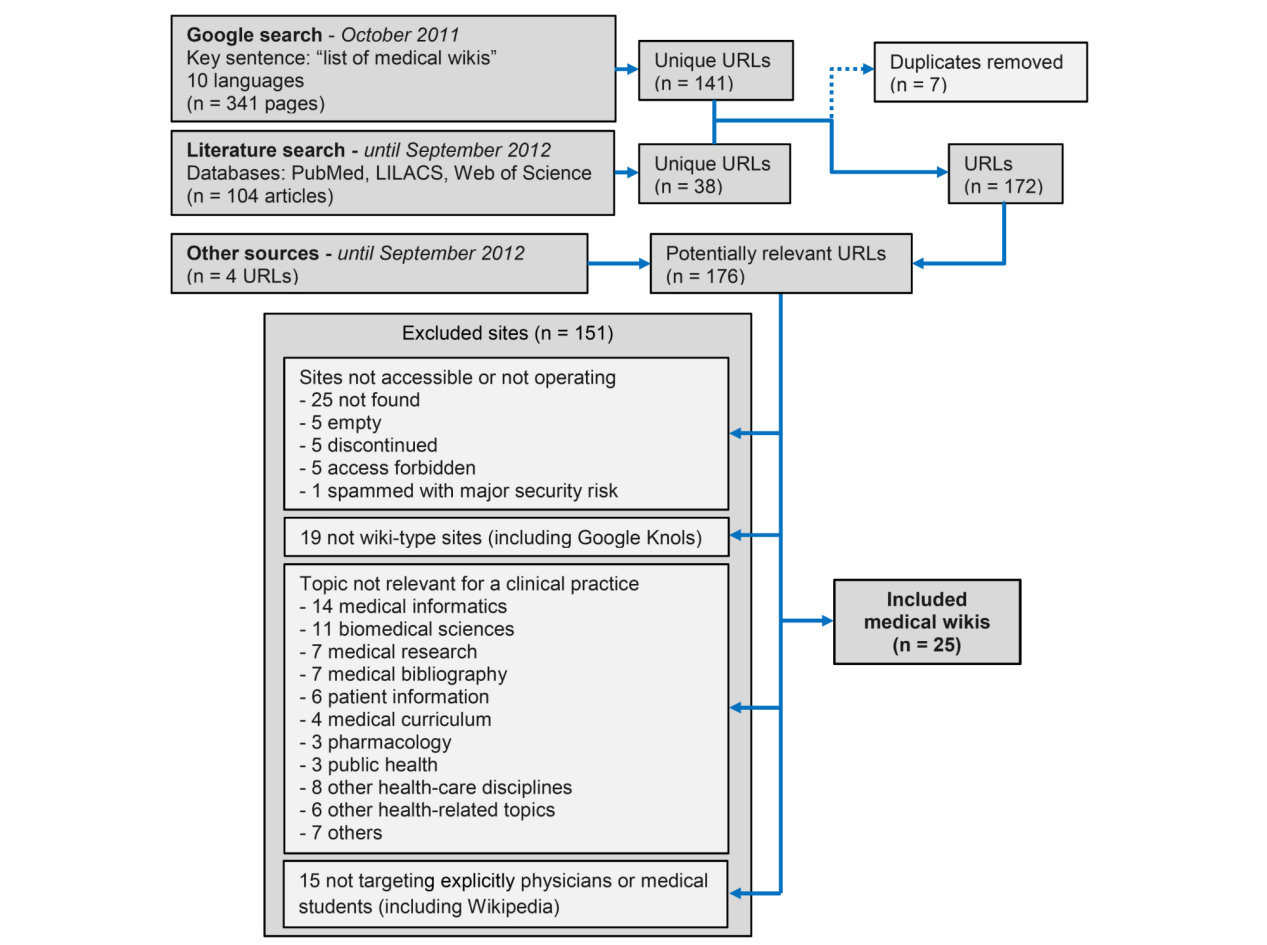
Site screening, exclusion, and inclusion flow diagram.

The Google search yielded 341 pages, including 27 linking to some potentially relevant URLs. After extraction and removing duplicates, 141 URLs were collected (Multimedia Appendix 1). The literature search yielded 133 articles, 104 after removing duplicates. After identification of potentially relevant URLs and removing duplicates, 38 URLs were collected (Multimedia Appendix 2). Four additional potentially relevant URLs were retrieved from other sources. Merging all results and removing duplicates, 176 potentially relevant URLs were finally collected (Figure 1, Multimedia Appendix 3).

### Sites’ Exclusion and Inclusion

Of the 176 collected URLs, 31 met the inclusion criteria. Six of them became inoperative during the study. Finally, 25 wikis were retained for analysis [35-59] (Figure 1; Multimedia Appendix 3).

### Sites’ Description and Assessment

#### Purposes

The main languages were English (19 wikis), German (3), French (2), and Chinese (1), and four wikis had a second language interface. The purpose was encyclopedic for 11 wikis, including one also aiming at reporting clinical cases. Among the 14 wikis having a non-encyclopedic purpose, five aimed at editing a textbook, three medical lessons, two oncology protocols, one a single focused article, and three at reporting clinical cases, including one also displaying a textbook-like wiki area. Whereas 16 wikis were specialized to specific themes or disciplines, nine were not. Physicians were explicitly targeted by 22 wikis, medical learners by 18, and lay persons by five (Table 2).

**Table 2:** Wikis’ purposes.

Wiki			Language	Main purpose(s)	Target audience
**Encyclopedic**					
		Medpedia [35]	English	Medical encyclopedia	Physicians, Learners, Laypeople
		Ganfyd [36]	English	Medical knowledge base	Physicians
		AskDrWiki [37]	English	Medicine	Physicians, Learners
		DocCheck Flexikon [38]	German, English	Medical lexicon	Physicians
		Toxipedia [39]	English, Spanish	Toxicology encyclopedia	Physicians, Learners
		EyeWiki [40]	English	Ophthalmology encyclopedia	Physicians, Learners
		Radiopaedia [41]	English	Radiology encyclopedia & clinical case reports	Physicians
		Wikiecho [42]	English	Echography encyclopedia	Physicians
		wikiRadiography [43]	English	Radiography resource	Physicians
		Pathowiki [44]	German	Pathology encyclopedia	Physicians, Learners
		Pathpedia [45]	English	Pathology wikibook	Physicians, Learners, Laypeople
**Non-Encyclopedic**					
	**Textbook**				
		WikiDoc [46]	English	Medical textbook	Physicians, Learners, Laypeople
		WardWiki [47]	English	Junior doctors help	Physicians, Learners
		WikEM [48]	English	Emergency Medicine point of care reference	Physicians, Learners
		Open Anesthesia [49]	English	Anesthesia textbook & critical care manual	Physicians, Learners
		ECGpedia [50]	English, Dutch	ECG textbook & tutorial	Physicians, Learners
	**Lessons**				
		MedRevise [51]	English	Medical course revision	Learners
		Mediwiki.fr [52]	French	Medical course revision	Learners
		Wikia Biomedwiki [53]	German	Bio-medical learning aid	Physicians, Learners
	**Protocols**				
		Oncologik [54]	French	Oncology protocols	Physicians
		OncoWiki [55]	English	Oncology regimens	Physicians
	**Single article**				
		Open Medicine Live Wiki [56]	English	Second line oral therapy in type 2 diabetes	Physicians, Learners, Laypeople
	**Clinical cases reports**				
		Dermpedia [57]	English	Dermatology knowledge and experience sharing	Physicians, Learners
		Orthochina [58]	Chinese, English	Orthopedic clinical cases	Physicians, Learners, Laypeople
		UCLA Radiology Residents Pediatric Imaging [59]	English	Radiology clinical cases	Learners

#### Platform

MediaWiki in its native form was supporting 15 sites. Three sites were hosted by online “wiki farms”, that are ready-to-use multifunctional platforms [60-62]. The remaining seven sites had purpose-built platforms, including two developed upon MediaWiki. As opposed to every purpose-built platform, only one site using MediaWiki natively systematically managed users’ real names and credentials. Wiki farms and purpose-built platforms included various forms of forums and social networks. Editing on MediaWiki required using a specific mark-up language, whereas all other software had a “What You See is What You Get” editing interface. Three wikis had automated links to PubMed or Cochrane library external databases. Two wikis operated a semantic management for synonyms or keywords. Two wikis provided some medical imaging facilities (Table 3).

**Table 3:** Wikis’ platform.

Wiki			Software	Purpose-built	User disclosures management	Relevant utilities^a^
**Encyclopedic**						
		Medpedia [35]	MediaWiki	✓	✓	
		Ganfyd [36]	MediaWiki			Bibl. links
		AskDrWiki [37]	MediaWiki			
		DocCheck Flexikon [38]	MediaWiki	✓	✓	
		Toxipedia [39]	Other	✓	✓	
		EyeWiki [40]	MediaWiki			
		Radiopaedia [41]	Other	✓	✓	Imaging + semantics
		Wikiecho [42]	MediaWiki			
		wikiRadiography [43]	Online wiki farm			
		Pathowiki [44]	MediaWiki			
		Pathpedia [45]	Other	✓	✓	
**Non-Encyclopedic**						
	**Textbook**					
		WikiDoc [46]	MediaWiki			Bibl. links + semantics
		WardWiki [47]	MediaWiki			
		WikEM [48]	MediaWiki		✓	
		Open Anesthesia [49]	MediaWiki			
		ECGpedia [50]	MediaWiki			
	**Lessons**					
		MedRevise [51]	MediaWiki			
		Mediwiki.fr [52]	MediaWiki			
		Wikia Biomedwiki [53]	Online wiki farm			
	**Protocols**					
		Oncologik [54]	MediaWiki			Bibl. links
		OncoWiki [55]	MediaWiki			
	**Single article**					
		Open Medicine Live Wiki [56]	MediaWiki			
	**Clinical case reports**					
		Dermpedia [57]	Other	✓	✓	
		Orthochina [58]	Other	✓	✓	Imaging
		UCLA Radiology Residents Pediatric Imaging [59]	Online wiki farm			

^a^Bibl. links=automatized links to external resources (PubMed, Cochrane, etc); Semantics=key words management; Imaging=medical imaging facilities.

#### Management

Sites’ owners were non-profit organizations (n=10), individuals (n=6), private companies (n=4), scientific societies (n=2) or universities (n=2), and one could not be identified. Six wikis restricted access to their talk pages and users’ profile areas, and one wiki restricted access to its articles. Two wikis allowed any visitor to edit without registering. Registration was automated in 11 wikis, based upon credentials in 10, and limited to a closed community in four. A hierarchy between registered users existed in 14 wikis, among which three restricted the edition (or the validation of edition proposals) to super-users only. Super-users could be organized in “editorial boards” (n=9), responsible for the whole content, in “lead authors” (n=4), responsible for some articles, or in “moderators” (n=2), responding on call. Super-users were nominated without any explicit procedure in 10 wikis, subjectively in consideration of users’ credentials or activity in two wikis, and following a systematic procedure based on a score or a vote in two wikis. Super-users were divided in more than two types of roles in four wikis (Table 4).

**Table 4:** Wikis’ management.

Wiki			Governance^a^	Edit right accreditation	Authoring structure	Super-users nomination	>2 super-user roles
**Encyclopedic**							
		Medpedia [35]	Universities	Super-uservonly	Lead authoring	On credentials^b^ + on score^c^	
		Ganfyd [36]	NPO	On credentials^b^	None	-	
		AskDrWiki [37]	NPO	Super-user only	Lead authoring	On credentials	✓
		DocCheck Flexikon [38]	PC	(any visitor)	None	-	
		Toxipedia [39]	NPO	Automated	Editorial board	N/A	
		EyeWiki [40]	SS	On credentials	Editorial board	N/A	
		Radiopaedia [41]	PC	Automated	Editorial board	N/A	
		Wikiecho [42]	NPO	Automated	Editorial board	N/A	
		wikiRadiography [43]	Individuals	Automated	Moderators	N/A	
		Pathowiki [44]	University	On credentials	None	-	
		Pathpedia [45]	PC	Automated	Editorial board	N/A	
**Non-Encyclopedic**							
	**Textbook**						
		WikiDoc [46]	NPO	On credentials	Editorial board	N/A	✓
		WardWiki [47]	N/A	Closed	-	-	
		WikEM [48]	NPO	Automated	Editorial board	On credentials + editorial activity	✓
		Open Anesthesia [49]	SS	Automated	Editorial board	N/A	
		ECGpedia [50]	NPO	On credentials	Lead authoring	N/A	
	**Lessons**						
		MedRevise [51]	Individuals	On credentials	None	-	
		Mediwiki.fr [52]	Individuals	On credentials	None	-	
		Wikia Biomedwiki [53]	Individual	(any visitor)	None	-	
	**Protocols**						
		Oncologik [54]	NPO	Closed	-	-	
		OncoWiki [55]	Individual	Closed	-	-	
	**Single article**						
		Open Medicine Live Wiki [56]	NPO	Automated	None	-	
	**Clinical case reports**						
		Dermpedia [57]	PC	Automated	Editorial board + lead authoring	N/A	
		Orthochina [58]	NPO	Super-user only	Moderators + editorial board	Automated + on score^d^ + vote	✓
		UCLA Radiology Residents Pediatric Imaging [59]	Individual	Closed	-	-	

^a^NPO=non-profit organization; PC=private company; SS=scientific society

^b^Proof of credentials required.

^c^Score based on forum contributions and edit proposals.

^d^Score based on a multiple choice test and forum contributions.

#### Information Framework

The owner’s identity was displayed on 19 wikis, its contact details on 21, its funding sources on 14, and its potential conflicts of interest on seven. A medical advisory statement was displayed on 17 wikis, a policy for users’ privacy on 17, and a policy about advertising on 10. A review policy was displayed on 10 wikis, a rule for the protection of patients’ data on 11, a rule for referencing information on nine, a rule for delivering true information on 11, and a rule for organizing content on five. The editing users’ identity was systematically displayed on nine wikis, their credentials on seven, their potential conflicts of interest on two, and the administrators’ identity was systematically displayed on three wikis, which were all made by students [51,52,59]. The total information framework quality score ranged from zero to 15 out of 16, with a median score of 6 (Table 5). The correlation between raters was fair (*R* ^2^=.68). Beyond these criteria, only one wiki organized standardized peer-reviews [39].

**Table 5:** Wikis’ information framework quality assessment.

Wiki			Owner disclosures (n=4)	Disclaimers (n=3)	Editorial policy (n=5)	User disclosures (n=4)	Total (n=16)
**Encyclopedic**							
		Medpedia [35]	4	3	5	3	15
		Ganfyd [36]	3	2	2	0	7
		AskDrWiki [37]	4	1	2	0	7
		DocCheck Flexikon [38]	3	2	0	0	5
		Toxipedia [39]	4	3	3	3	13
		EyeWiki [40]	4	3	3	0	10
		Radiopaedia [41]	4	3	4	2	13
		Wikiecho [42]	2	2	1	0	5
		wikiRadiography [43]	0	2	0	0	2
		Pathowiki [44]	3	2	3	0	8
		Pathpedia [45]	4	2	3	2	11
**Non-Encyclopedic**							
	**Textbook**						
		WikiDoc [46]	4	3	5	0	12
		WardWiki [47]	0	2	3	0	5
		WikEM [48]	2	1	0	3	6
		Open Anesthesia [49]	3	0	2	0	5
		ECGpedia [50]	3	2	2	0	7
	**Lessons**						
		MedRevise [51]	3	2	2	1	8
		Mediwiki.fr [52]	2	1	1	1	5
		Wikia Biomedwiki [53]	0	2	0	0	2
	**Protocols**						
		Oncologik [54]	2	0	1	1	4
		OncoWiki [55]	1	1	0	0	2
	**Single article**						
		Open Medicine Live Wiki [56]	0	0	0	0	0
	**Clinical case reports**						
		Dermpedia [57]	3	2	2	2	9
		Orthochina [58]	1	2	1	2	6
		UCLA Radiology Residents Pediatric Imaging [59]	2	1	1	1	5

#### Contributions

Physicians were considered as contributors by default in all wikis except the three made by and for students [51,52,59]. Medical learners contributed according to a formal educational goal on four wikis, and as super-users on five wikis. Lay persons contributed to four wikis. Clinical cases were reported on nine wikis (Table 6).

**Table 6:** Wikis’ contributions.

Wiki			Lay people	Learners^a^	Formal educational goal	Clinical case reports
**Encyclopedic**						
		Medpedia [35]				✓
		Ganfyd [36]				
		AskDrWiki [37]				
		DocCheck Flexikon [38]	Free edition			
		Toxipedia [39]	Registered only			
		EyeWiki [40]				
		Radiopaedia [41]				✓
		Wikiecho [42]				
		wikiRadiography [43]				✓
		Pathowiki [44]		PG		✓
		Pathpedia [45]				
**Non-Encyclopedic**						
	**Textbook**					
		WikiDoc [46]				✓
		WardWiki [47]				
		WikEM [48]		PG	✓	
		Open Anesthesia [49]		PG	✓	
		ECGpedia [50]		PG		✓
	**Lessons**					
		MedRevise [51]		UG		✓
		Mediwiki.fr [52]		UG + PG		
		Wikia Biomedwiki [53]	Free edition	UG		
	**Protocols**					
		Oncologik [54]				
		OncoWiki [55]				
	**Single article**					
		Open Medicine Live Wiki [56]	Registered only			
	**Clinical case reports**					
		Dermpedia [57]				✓
		Orthochina [58]		CME	✓	✓
		UCLA Radiology Residents Pediatric Imaging [59]		PG	✓	✓

^a^UG=undergraduate, PG=postgraduate, CME=practicing physicians in continuing medical education.

#### Content

As only one wiki displayed a single article and another did not allow access to its relevant content, 116 articles were sampled, including 58 most revised and 58 most finalized. Numbers of authors were not available for five encyclopedic articles. Numbers of revisions and of authors were not available for five non-encyclopedic articles. Pictures, videos, and external resources were more frequent in articles from encyclopedic wikis. Posology details were more frequent in articles from non-encyclopedic wikis (*P*<.01). The Flesch reading ease scores were lower in encyclopedic wikis (Table 7).

**Table 7:** Features of content, of edition, and readability of articles according to wiki purpose (N=116 articles).

Wiki purpose		Encyclopedic (n=55)	Non-encyclopedic (n=61)	*P* value
		n (%) or median (min-max)	n (%) or median (min-max)	
**Content**				
	Pictures, n (%)	33 (60.0)	23 (37.7)	.025
	Videos, n (%)	7 (12.7)	0 (0.0)	.004
	Diagrams, n (%)	3 (5.5)	8 (13.1)	.211
	Posology, n (%)	5 (9.1)	24 (39.3)	< .001
	Evidence levels, n (%)	0 (0.0)	2 (3.3)	.497
	External resources, n (%)	33 (60.0)	21 (34.4)	.009
	References, median (min-max)	3 (0-87)	2 (0-105)	.400
	Words, median (min-max)	1248 (94-4945)	654 (38-16265)	.353
**Edition, median (min-max)**				
	Revisions	40 (2-261)	40.5 (2-516)	.953
	Authors	3 (1-34)	3 (1-6)	.067
	Talks	0 (0-24)	0 (0-2)	.099
**Readability, median (min-max)**				
	Flesch’s reading ease score	26.1 (-11.4-50.6) (college graduate)	33.9 (-55.5-87.6) (college)	.041

#### Activities

Wikis had been created between 1997 and 2011 (median year: 2007). Content pages per wiki varied from 3 to 98,039 (median 620.5), revisions per content page from 3.6 to 180.5 (median 17.7), and talk pages per content page from 0 to 0.42 (median 0.015). Among five particularly active wikis, three had a high previous year editorial rate and three a sharply increasing editorial trend. Among six wikis almost unused, six had a low previous year editorial rate, and three a sharply decreasing editorial trend. The activity of one wiki having a sharply increasing trend upon a very low previous editorial rate was not interpreted (Table 8). Two wikis included in this review were discontinued after the completion of the study [35,47].

**Table 8:** Wikis’ activities.

Wiki			Year of creation	Content pages	Revisions / content pages	Talk pages / content pages	2011-12 editorial rate^a,b^, %	2010-12 editorial trend^a,c^
**Encyclopedic**								
		Medpedia [35]	2002	4000	85.3	0.02	36	→
		Ganfyd [36]	2005	7979	6.7	0.14	18	→
		AskDrWiki [37]	2006	1406	4.8	< 0.01	4	→
		DocCheck Flexikon [38]	2002	18,017	8.5	0.02	73	↗
		Toxipedia [39]	2006	1910^a^	N/A	N/A	34	→
		EyeWiki [40]	2010	142	79.2	0.20	41	→
		Radiopaedia [41]	2005	5131	N/A	N/A	44	→
		Wikiecho [42]	2007	N/A	N/A	N/A	N/A	N/A
		wikiRadiography [43]	2006	1730^a^	N/A	N/A	10	↘
		Pathowiki [44]	2010	425	11	< 0.01	27	→
		Pathpedia [45]	2006	N/A	N/A	N/A	0	N/A
**Non-Encyclopedic**								
	**Textbook**							
		WikiDoc [46]	2006	98,039	6.7	< 0.01	38	↗
		WardWiki [47]	2010	324	11.4	0	0	↘
		WikEM [48]	2010	126	N/A	0.01	64	→
		Open Anesthesia [49]	2008	1023	N/A	0.02	60	→
		ECGpedia [50]	2006	1241	17.7	0.02	16	→
	**Lessons**							
		MedRevise [51]	2008	597	21.8	0.01	8	→
		Mediwiki.fr [52]	2008	216	29.4	0.02	32	↗
		Wikia Biomedwiki [53]	2006	75	36.0	0.02	4	↗
	**Protocols**							
		Oncologik [54]	2011	152	180.5	0.42	48	→
		OncoWiki [55]	2011	112	3.6	0.01	N/A	N/A
	**Single article**							
		Open Medicine Live Wiki [56]	2011	3	29.0	0	0	↘
	**Clinical case reports**							
		Dermpedia [57]	2008	601	N/A	N/A	18	→
		Orthochina [58]	1997	N/A	N/A	N/A	N/A	N/A
		UCLA Radiology Residents Pediatric Imaging [59]	2008	640	N/A	0	17	→

^a^Estimated with Google.

^b^[Last year edited pages]/[total pages]: >50%=high rate; <10%=low rate

^c^[Last year edited pages ]/[year before edited pages]: ↗=sharply increasing trend (>300 %); ↘=sharply decreasing trend (<33 %); →=stable trend.

## Discussion

### Principal Findings

From this international review, we identified 25 medical wikis dedicated to clinical practices. The majority were in English and four were bilingual. They had various purposes, dominated by encyclopedic perspectives (44%), and most were specialized (64%). The MediaWiki software was commonly used (68%), often in its native form (60%). Site owners were mostly non-profit organizations (40%) and individuals (24%); only two were universities. While practicing physicians were major contributors (88%), medical learners (36%) and lay persons (16%) sometimes contributed.

Cross-reading our results, the relevancy for clinicians of the medical wikis can be discussed according to four information properties: accuracy, readability, reliability, and currency. Accuracy may be impaired in wikis not displaying a review policy (60%) and in those not delivering rules for organizing content (80%) [63,64]. The articles from encyclopedic wikis presented characteristics less relevant for professional use than the others, including more pictures, videos, and external resources but fewer posology details. The Flesch reading ease scores were globally low, especially for encyclopedic articles. In regard to reliability, 64% of wikis fulfilled less than half of the information framework quality criteria. In addition, articles were poorly referenced, and evidence level notifications were exceptional. Finally, 88% of the wikis had fewer than 50% of articles revised in the last year, and 24% of the sites were almost unused.

### Strengths and Limitations

Our review may not have been exhaustive as the Google search was restricted to lists of medical wikis and several sites reported in the health literature were not accessible. Furthermore, the Web 2.0 field is rapidly changing, and some new medical wikis may have emerged since October 2012. Re-browsing the lists of medical wikis used in this study, we found only one relevant wiki after the inclusion period: the Australian Cancer Guidelines Wiki [65]. Among the 25 included sites, Medpedia and WardWiki have been discontinued [35,47], and a few changes occurred in the structure of the others: Open Anesthesia has been reorganized [49], WikiEcho and MedRevise changed their “skin” [42,51], and Oncologik added a missing link to its owner [54].

Among the tools available for assessing the quality of health information on websites, none is currently validated and none is fitted either to wikis or to a professional audience [66,67]. The HSWG IQ tool does not take into account collaborative features, as acknowledged by Dobrogowska-Schlebusch [15], and it has been removed from the Web [19]; the DISCERN tool targets health consumers and is restricted to information on treatments [68]; and the Bomba and Land Index has also been designed for health consumers [69]. Numerous items are common between these questionnaires and major guidelines such as the eHealth code of ethics [70], the American Medical Association guideline [71], or the eEurope 2002 quality criteria [72]. The HONcode ethical code of conduct is unique to provide specifications for collaborative websites [31,73]. For example, the item “is the information referenced?” will be transposed for collaborative websites as “is there a statement asking platform users to give references to the information they provide?”. Such specifications do not directly apply to the content, but indirectly through the editorial framework. However, the right influence of the framework on the content deserves to be investigated in future research projects.

The relevancy of low readability scores, corresponding to college and higher, is arguable since medical doctors have *de facto* a high level of reading. It has been long demonstrated that readability impacts both the understanding and the cross-reading ability, even for highly educated readers [74], and the need for simplicity is expressed by clinicians themselves for practice guidelines [4]. The relevancy of the Flesch reading ease test for medical writings is also debatable, but more specific tools are not yet validated [75]. Although it includes adjustment parameters adapted to several languages [34], a linguistic bias cannot be excluded in this study since multilingual comparisons have not been documented.

To check the validity of the estimation of annual editorial activities using Google, we measured the agreement between the number of content pages declared on the site and the corresponding estimate from the Google search engine, for 20 wikis. Although there was a strong agreement (Spearman correlation coefficient=.88, *P*<.001), automated page creation and vandalism may bias both figures.

### Unmet Clinical Needs

Our results suggest that no medical wiki meets all four information properties needed by clinicians. The encyclopedic format does not seem to fit in terms of both accuracy and readability. However, whatever the wikis’ purposes, the organization of contents is often unclear, apart from very focused purposes such as oncology protocols, where the knowledge granularity is adapted to a particular audience [54]. The Medical Subject Headings (MeSH) indexing system is sometimes integrated, but it requires specific training for contributors, which is challenging in a multi-authoring context [76]. Whereas some semantic utilities can help manage indexation constraints [10,77], add-ons aimed at improving either medical knowledge management or ergonomics are rarely implemented in medical wikis. If such gaps impact both accuracy and readability, they may also hamper the involvement of users. Contrary to pure knowledge content, the frequent clinical case reports in medical wikis, supporting the emergence of concrete questions of practice, are likely to meet strong clinical interest.

Reliability is widely, and sometimes critically, impaired by lack of management. Although authoring transparency requires both technical and policy supports [5], our framework assessment particularly shows gaps in users’ disclosures and editorial policies. Since almost only purpose-built platforms are able to manage detailed user data, technical issues are important. Among open communities, only 48% of medical wikis ask for credentials to register, with two requiring some proof [35,36]. As an alternative, users’ medical skills can be assessed during an automated registration including medical tests [58,78]. Interestingly, the fully opened Wikipedia’s articles are commonly consulted by clinicians and medical students [79], while their relevancy has been recurrently questioned [7,14,16-18,21]. However, Wikipedia, including its Wikiproject Medicine, cannot respond to specific clinical needs as it does not target any specific audience [28]. As an encyclopedic media, it is also likely to meet the limitations highlighted in this study.

In most wikis, weak and poorly collaborative activity jeopardizes content updates. The talk pages, when available, are exceptionally used, and the discussion threads included in forums or social networks are not directly connected to content pages [80]. As a consequence, adversarial debates are lacking, although they are a foundation for building evidence [3].

### The Open Community Challenge

Users’ regulation in wikis is complex since the lower the control of their editors, the higher their growth [81]. For example, Wikipedia’s English article on atrial fibrillation has been revised approximately 1345 times and discussed 150 times [82], and the article on the recent drug dabigatran 555 times and 35 times respectively [83]. Apart from the severe reliability issues due to anonymity in Wikipedia [84], it has been shown that its development, based only on volunteering, leads articles to be unevenly readable, complete, and reliable [17,20,85]. In our study, we paradoxically observed the highest page revision and discussion levels in a small wiki reserved to a closed community [54]. This finding suggests that a strong user commitment can overcome volunteering limitations.

Although multi-authoring requires a thorough organization [86], communities attached to medical wikis are often poorly structured. Super-user nominations are usually opaque, and only one wiki provides a standardized peer-review process [39]. As implemented in two wikis, the extent of users’ rights can depend on their participation level [35,58], which represents a reward for authors [87]. However, in order to open scientific debates, the organization of bottom-up relations between users should be further considered [88]. In this way, the public expertise promoted by Wikipedia, which is based on consensus, uses a complex and democratic moderation system, detailed editorial rules, and standardized peer-reviews [21].

While the HONcode principle about the authoritativeness of the information protects the moderators’ privacy by allowing their anonymity [31], it cannot guarantee the trustworthiness of what they have written [84,89]. The professional scope of our review highlights a lack of audience specifications in health information quality initiatives, in particular for collaborative applications where readers and writers are mixed altogether. The extensive review of social media by Grajales et al provides a useful tutorial for health care professional end users, which may be a first step to building more detailed guidelines for professional health information on the Internet [7]. Indeed, some professional knowledge may generate adverse outcomes, as information on drugs with potential for misuse is commonly sought on the Internet [90]. Therefore, as included in the wikiproject Medicine of Wikipedia [21], a policy specifying the nature and the limits of publicly accessible content is critical, and a model for displaying health information is needed [67,73].

### Educational Value Added

Among eight medical wikis including learners’ contributions, five include spontaneous undergraduate or postgraduate students’ contributions. The three others have a formal educational goal, targeting postgraduate students or practicing physicians in continuous medical education [49,58,59]. Educational goals may represent an alternative to mere volunteering since learners’ contributions can be part of their curricula. As works performed in training are frequently based on clinical cases as starting points for gathering scientific evidence [91,92], the wiki principle seems particularly fitted to archive, share, discuss, and gradually improve the related materials [93]. From a theoretical point of view, the wiki medium, as an asynchronous communication tool, embodies learning principles based on constructivism and cooperation [94]. Nevertheless, if Internet-based educational programs can be an alternative to live interactive workshops [95], the effectiveness of collaborative writing applications in medical education requires further research [12,14].

### Conclusions

The 25 medical wikis reviewed present various limitations in their format, management, and collaborative features. Encyclopedic wikis have less accurate and readable content. Reliability is widely impaired by lack of transparency. Currency is commonly jeopardized by low editorial activity. Professional medical wikis may be improved by using clinical cases, developing more detailed transparency and editorial policies, and involving postgraduate and continuing medical education learners.

## Multimedia Appendix 1

Google search.

## Multimedia Appendix 2

Literature search.

## Multimedia Appendix 3

Site exclusions and inclusions.

## Abbreviations

CMS: Content Managing System
HONcode: Health On the Net code of ethics
HSWG IQ tool: Health Summit Working Group Information Quality tool
MeSH: Medical Subject Headings
URL: Uniform Resource Locator
