# Supplementary material for: Medical Wikis Dedicated to Clinical Practice: A Systematic Review
Source: J Med Internet Res. 2015 Feb 19;17(2):e48. doi: 10.2196/jmir.3574 (PMC4392552; doi:10.2196/jmir.3574)
Supplement: Supplementary file 1 [file jmir_v17i2e48_app1.pdf]

Appendix 1 : Google search

1. Google queries

Language	Search criteria	Date	Resulting pages	Relevant pages
English	*"list of medical wikis"*	*04/10/11*	*20,500*	2
English	"list of medical wikis" -davidrothman -rothman	04/10/11	62	12
Chinese simpl.	"医疗维基"	04/10/11	12	1
Chinese trad.	"醫療維基"	04/10/11	23	0
Spanish	lista+"wikis médicos"	04/10/11	54	6
Japanese	"医療ウィキ" +"のリスト"	04/10/11	88	1
French	liste+"wikis médicaux"	04/10/11	25	4
Portuguese	lista+"wiki médicos"	04/10/11	20	0
German	liste+"medizinischer wikis"	04/10/11	4	1
Arabic	قائمة + الويكيات + الطبية	04/10/11	34	0
Russian	список+"медицинские вики"	04/10/11	6	0
Korean	"의료 위키"+목록	04/10/11	12	0
**Total**			**341**	**27**

2. Google results despoliation

	Relevant pages (lists of medical wikis, or wikis themselves)	Language	Last update	Potentially relevant URLs displayed
1	davidrothman.net : List of Medical Wikis	En	21/01/09	57
2	davidrothman.net : More Health Information Wikis	En	07/08/08	7
3	Wikipedia : Category:Encyclopedias on medicine	En	24/12/10	7
4	Wikipedia : Online medical wiki encyclopedias	En	23/08/11	7
5	Healthplusplus	En	20/01/11	42
6	Dr Penna	En	29/11/09	9
7	P2pfondation : Medical Knowledge Sharing	En	25/02/10	8
8	ScienceRoll	En	27/03/07	29
9	HLWIKI : Top 50 Medical Wikis	En	11/09/11	52
10	HLWIKI : Medical wikis	En	18/06/11	39
11	HLWIKI : Top Web 2.0 Services in Medicine – 2011	En	11/09/11	8
12	HLWIKI : Health librarianship pathfinder	En	15/07/11	5
13	Wikiindex category:medical	En	01/09/08	50
14	Cutting-edge Web Resources for PT	En	19/08/09	12
15	WJKB	Ch	Na	1
16	Medicina Basada en la Evidencia y Web 2.0	Es	2008	12
17	INTERNET, PEDIATRIA Y LA WEB 2.0	Es	23/11/07	6
18	Las Tecnologías de la Información y Comunicación (TIC) y la formación continuada: las nuevas herramientas de Web 2.0	Es	Na	1
19	PAMI CAPACITACION : web2.0	Es	13/07/11	5
20	uroportal.net : wikis	Es	29/09/11	6
21	Web 2.0 y la medicina	Es	02/12/08	4
22	Hibakuiryo	Jp	Na	1
23	JC | Blog	Fr	27/11/07	2

Appendix 1 : Google search

24	Médecine et Web 2.0 : wikis médicaux	Fr	20/08/09	8
25	Internet et ophtalmo : Lettre ouverte : médecine et Web 2.0	Fr	13/11/07	1
26	Santexel : catalogue 2011	Fr	04/09/11	5
27	blog.doccheck.com : Medical-Wikimania	De	11/06/07	5
	Total			389
	**Total after duplicates removed**			**141**
