# Supplementary material for: Medical Wikis Dedicated to Clinical Practice: A Systematic Review
Source: J Med Internet Res. 2015 Feb 19;17(2):e48. doi: 10.2196/jmir.3574 (PMC4392552; doi:10.2196/jmir.3574)
Supplement: Supplementary file 2 [file jmir_v17i2e48_app2.pdf]

Appendix 2: Literature search

Engine	Search criteria (full text)	Date	N articles	Wiki(s) description	Identified URLs
Pubmed	wiki AND (medic* OR clinic*)	01/10/12	73	54	33
Web of Science	wiki AND (medic* OR clinic*)	01/10/12	51	36	16
Lilacs	wiki	01/10/12	9	6	2
Total			133	96	51
**Total (after duplicates removed)**			**104**	**72**	**38**
