# Supplementary material for: Medical Wikis Dedicated to Clinical Practice: A Systematic Review
Source: J Med Internet Res. 2015 Feb 19;17(2):e48. doi: 10.2196/jmir.3574 (PMC4392552; doi:10.2196/jmir.3574)
Supplement: Supplementary file 3 [file jmir_v17i2e48_app3.pdf]

Appendix 3 : Sites exclusions and inclusions

N<br>176	Site<br>(25 included)	Access<br>(35 excl.)	Type<br>(19 excl.)	Topic<br>(76 excl.)	Audience<br>(15 excl.)	Status<br>(6 excl.)
1	Globus MEDICUS	discontinued				
2	Scitopics	discontinued				
3	Zyprexa	discontinued				
4	Student Clinics Wiki	empty				
5	Wikinvestigacion	empty				
6	Wikiprotein	empty				
7	Case School of Dental Medicine Clinic Manual	forbidden				
8	ECCLES HSL wiki	forbidden				
9	Epic Systems Corporation's Community Library	forbidden				
10	Just the Facts wiki	forbidden				
11	Partners Healthcare eRooms	forbidden				
12	APF, ASCP and PRODS wiki	not found				
13	ArrayWiki	not found				
14	AutismAspergersWiki	not found				
15	BillingWiki	not found				
16	BioKb	not found				
17	Community Huntington’s Disease Community Notebook	not found				
18	Efficient MD Wiki	not found				
19	eHealth Wiki (JMIR)	not found				
20	Healtheva	not found				
21	HerzKinderWiki	not found				
22	JournalReview.org	not found				
23	MacSurgWiki	not found				
24	Medical Practice Billing and Compliance	not found				
25	Medical University of South Carolina Department of Radiology’s Medical physics wiki	not found				
26	MedTech Wiki	not found				
27	PeacefulBeginnings	not found				
28	PubDrug	not found				
29	Student Doctor Network Wiki	not found				
30	TheRemedePointOrgWiki	not found				
31	WebHealth	not found				
32	Wiki des recommandations médicales	not found				
33	WikiAdvocate	not found				
34	WikiLiver	not found				
35	WiserWiki	not found				
36	Medicalopedia.org	Y	Blog			

Appendix 3 : Sites exclusions and inclusions

N<br>176	Site<br>(25 included)	Access<br>(35 excl.)	Type<br>(19 excl.)	Topic<br>(76 excl.)	Audience<br>(15 excl.)	Status<br>(6 excl.)
37	WikiBirdFlu	Y	Blog			
38	CIC Quinze-Vingt : études en cours	Y	CMS			
39	Consumer Health Information (Toronto Public Library)	Y	CMS			
40	Flu Wikie	Y	CMS			
41	InfoMedMD	Y	CMS			
42	Medcyclopaedia	Y	CMS			
43	MedSkills wiki project	Y	CMS			
44	Plant antiVenom	Y	CMS			
45	SmpaWiki	Y	CMS			
46	Wikinu Médecine	Y	CMS			
47	WikiPremed MCAT Course	Y	CMS			
48	AspiesForFreedom	Y	forum			
49	Sermo	Y	forum			
50	Videnbasen for Tourette Syndrom	Y	forum			
51	WikiHealthCare	Y	forum			
52	Google's Knol	Y	other			
53	HealthMash	Y	other			
54	Wikia : Wikia Category: Health	Y	other			
55	BRL-CAD wiki	Y	Wiki	Informatics		
56	Clinfowiki	Y	Wiki	Informatics		
57	Clinical Research Informatics Wiki	Y	Wiki	Informatics		
58	COMKAT	Y	Wiki	Informatics		
59	Gnumed	Y	Wiki	Informatics		
60	HealthGrid Wiki	Y	Wiki	Informatics		
61	Iphylo	Y	Wiki	Informatics		
62	KnowWE	Y	Wiki	Informatics		
63	OpenEMR Documentation Wiki	Y	Wiki	Informatics		
64	openMRS	Y	Wiki	Informatics		
65	Pathology Informatics wiki	Y	Wiki	Informatics		
66	saferEHR	Y	Wiki	Informatics		
67	TOPSAN	Y	Wiki	Informatics		
68	Wikia : Biomedical Cybernetics Wiki	Y	Wiki	Informatics		
69	age-phenome-wiki	Y	Wiki	Fundamental sciences		
70	CHDwiki	Y	Wiki	Fundamental sciences		
71	conceptwiki - wikiproteins	Y	Wiki	Fundamental sciences		
72	Fuse	Y	Wiki	Fundamental sciences		
73	Harvester Wiki	Y	Wiki	Fundamental sciences		

Appendix 3 : Sites exclusions and inclusions

N<br>176	Site<br>(25 included)	Access<br>(35 excl.)	Type<br>(19 excl.)	Topic<br>(76 excl.)	Audience<br>(15 excl.)	Status<br>(6 excl.)
74	OpenWetWare	Y	Wiki	Fundamental sciences		
75	NeuroAnatomy	Y	Wiki	Fundamental sciences		
76	Pamela Stanley Lab Wiki	Y	Wiki	Fundamental sciences		
77	SilicoTryp wiki	Y	Wiki	Fundamental sciences		
78	Snpedia	Y	Wiki	Fundamental sciences		
79	WikiGenes	Y	Wiki	Fundamental sciences		
80	Urgencyclopedie	Y	Wiki	First aid		
81	Wikia : Street Medic Wikia	Y	Wiki	First aid		
82	NAHRS/MLA Nursing Resources wiki	Y	Wiki	Nursing		
83	Nursing Wiki	Y	Wiki	Nursing		
84	Neuromuscular PT Evidence	Y	Wiki	Physiotherapy		
85	Physiopedia	Y	Wiki	Physiotherapy		
86	The Lenore Thomson Exegesis Wiki	Y	Wiki	Psychology		
87	Wikia : Psychology wiki	Y	Wiki	Psychology		
88	ADEpedia	Y	Wiki	Medical research		
89	EBHC Search Strategies Wiki	Y	Wiki	Medical research		
90	Family Medicine Research wiki	Y	Wiki	Medical research		
91	Medical Images Wiki	Y	Wiki	Medical research		
92	Neurodegeneration Research Wiki	Y	Wiki	Medical research		
93	OBI Wiki	Y	Wiki	Medical research		
94	Wikia : Quality of Medical Data	Y	Wiki	Medical research		
95	e-Meducation	Y	Wiki	Medical bibliography		
96	EBM Librarian	Y	Wiki	Medical bibliography		
97	Médecine et Web 2.0	Y	Wiki	Medical bibliography		
98	Medical Matters Wiki - University south alabama	Y	Wiki	Medical bibliography		
99	MLA-HLS Wiki	Y	Wiki	Medical bibliography		
100	palliative care resources for palliative physician assistant	Y	Wiki	Medical bibliography		
101	Wiki des Bibliothèques Universitaires de Médecine et Santé Publique – Lausanne	Y	Wiki	Medical bibliography		
102	International Aids Vigil, Toronto	Y	Wiki	Patient information		
103	Muko-wiki	Y	Wiki	Patient information		
104	Wikia : Autism Wiki	Y	Wiki	Patient information		
105	Wikia : Cancer Help Infosite	Y	Wiki	Patient information		
106	Wikia : Celiac Wiki	Y	Wiki	Patient information		
107	WikiCancer	Y	Wiki	Patient information		
108	Medical Education Home	Y	Wiki	Medical curriculum		
109	Mediwiki	Y	Wiki	Medical curriculum		

Appendix 3 : Sites exclusions and inclusions

N<br>176	Site<br>(25 included)	Access<br>(35 excl.)	Type<br>(19 excl.)	Topic<br>(76 excl.)	Audience<br>(15 excl.)	Status<br>(6 excl.)
110	UML - Anesthesia Clinical Assistants Program	Y	Wiki	Medical curriculum		
111	WikiTox	Y	Wiki	Medical curriculum		
112	PharmLib	Y	Wiki	Pharmacology		
113	RxWiki	Y	Wiki	Pharmacology		
114	Wikia : DrogenWiki	Y	Wiki	Pharmacology		
115	Fluwiki	Y	Wiki	Public Health		
116	McGill Library Global Health Resource Guide	Y	Wiki	Public Health		
117	MIGHEALTHNET	Y	Wiki	Public Health		
118	Health++	Y	Wiki	Health-related		
119	HLWIKI Canada	Y	Wiki	Health-related		
120	Rife Wiki	Y	Wiki	Health-related		
121	Wellness Wiki	Y	Wiki	Health-related		
122	Wikia : Pet Diabetes Wiki	Y	Wiki	Health-related		
123	WikiHealth	Y	Wiki	Health-related		
124	Charity Scorecard	Y	Wiki	Other		
125	Citizendium	Y	Wiki	Other		
126	Knowledge Unifying Initiator	Y	Wiki	Other		
127	WikiIndex	Y	Wiki	Other		
128	Wikilearning	Y	Wiki	Other		
129	Wikimedia : Wikisource	Y	Wiki	Other		
130	WJBK	Y	Wiki	Other		
131	AIDS Wiki	Y	Wiki	Medicine	Other	
132	atwiki/hibakuiryo (medical radiation@wiki)	Y	Wiki	Medicine	Other	
133	Clusterkopfschmerz: CK-Wissen	Y	Wiki	Medicine	Other	
134	Ferato	Y	Wiki	Medicine	Other	
135	HivWiki	Y	Wiki	Medicine	Other	
136	Psywiki.org	Y	Wiki	Medicine	Other	
137	surgwiki	Y	Wiki	Medicine	Na	
138	Wikia : Diabetes Wiki	Y	Wiki	Medicine	Other	
139	Wikia : Flu Wiki	Y	Wiki	Medicine	Other	
140	Wikia : Medical Imaging	Y	Wiki	Medicine	Other	
141	Wikia : Wikianswers Doctors	Y	Wiki	Medicine	Other	
142	Wikimedia : Wikibooks : Health sciences	Y	Wiki	Medicine	Other	
143	Wikimedia : WikiMedia Commons – Medicine	Y	Wiki	Medicine	Other	
144	Wikimedia : Wikipedia : Medicine Portal	Y	Wiki	Medicine	Other	
145	Wikimedia : Wikiversity - School:Medicine	Y	Wiki	Medicine	Other	
146	QTinterval Wiki	Y	Wiki	Medicine	Phys.	Discontinued
147	RadsWiki	Y	Wiki	Medicine	Phys.	Discontinued

Appendix 3 : Sites exclusions and inclusions

N<br>176	Site<br>(25 included)	Access<br>(35 excl.)	Type<br>(19 excl.)	Topic<br>(76 excl.)	Audience<br>(15 excl.)	Status<br>(6 excl.)
148	Radiology Wiki	Y	Wiki	Medicine	Phys.	Empty
149	Wikisurgery	Y	Wiki	Medicine	Phys.	Empty
150	KidneyWiki	Y	Wiki	Medicine	Phys., stud.	Not found
151	WikiMD	Y	Wiki	Medicine	Phys., stud.	Spammed
152	AskDrWiki	Y	Wiki	Medicine	Phys., stud.	Operating
153	DocCheck Flexikon	Y	Wiki	Medicine	Phys.	Operating
154	Dermpedia	Y	Wiki	Medicine	Phys., stud.	Operating
155	ECGpedia	Y	Wiki	Medicine	Phys., stud.	Operating
156	EyeWiki	Y	Wiki	Medicine	Phys.	Operating
157	Ganfyd	Y	Wiki	Medicine	Phys.	Operating
158	Mediwiki.fr	Y	Wiki	Medicine	Stud.	Operating
159	Medpedia	Y	Wiki	Medicine	Phys.	Operating
160	MedRevise	Y	Wiki	Medicine	Stud.	Operating
161	Onco Wiki	Y	Wiki	Medicine	Phys., stud.	Operating
162	Oncologik	Y	Wiki	Medicine	Phys.	Operating
163	Open Anesthesia	Y	Wiki	Medicine	Phys., stud.	Operating
164	Open Medicine	Y	Wiki	Medicine	Phys.	Operating
165	Orthochina	Y	Wiki	Medicine	Phys.	Operating
166	Pathowiki	Y	Wiki	Medicine	Phys.	Operating
167	Pathpedia – wikibook	Y	Wiki	Medicine	Phys.	Operating
168	Radiopaedia	Y	Wiki	Medicine	Phys.	Operating
169	Toxipedia	Y	Wiki	Medicine	Phys.	Operating
170	UCLA Radiology Residents	Y	Wiki	Medicine	Stud.	Operating
171	WardWiki	Y	Wiki	Medicine	Phys., stud.	Operating
172	Wikia : Biomedwiki	Y	Wiki	Medicine	Phys., stud.	Operating
173	WikiDoc	Y	Wiki	Medicine	Phys.	Operating
174	WikiEcho	Y	Wiki	Medicine	Phys.	Operating
175	WikiEM	Y	Wiki	Medicine	Phys., stud.	Operating
176	WikiRadiography	Y	Wiki	Medicine	Phys.	Operating
